# Biotechnological Evolution of siRNA Molecules: From Bench Tool to the Refined Drug

**DOI:** 10.3390/ph15050575

**Published:** 2022-05-05

**Authors:** Danielle de Brito e Cunha, Ana Beatriz Teixeira Frederico, Tamiris Azamor, Juliana Gil Melgaço, Patricia Cristina da Costa Neves, Ana Paula Dinis Ano Bom, Tatiana Martins Tilli, Sotiris Missailidis

**Affiliations:** 1 Immunological Technology Laboratory, Institute of Technology in Immunobiologicals, Bio-Manguinhos, Oswaldo Cruz Foundation, Fiocruz, Rio de Janeiro 21040-900, Brazil; danielle.cunha@bio.fiocruz.br (D.d.B.e.C.); ana.frederico@bio.fiocruz.br (A.B.T.F.); tamiris.azamor@bio.fiocruz.br (T.A.); juliana.melgaco@bio.fiocruz.br (J.G.M.); pcristina@bio.fiocruz.br (P.C.d.C.N.); adinis@bio.fiocruz.br (A.P.D.A.B.); sotiris.missailidis@bio.fiocruz.br (S.M.); 2 Translational Oncology Platform, Center for Technological Development in Health, Oswaldo Cruz Foundation, Fiocruz, Rio de Janeiro 21040-900, Brazil; 3 Laboratory of Cardiovascular Research, Oswaldo Cruz Foundation, Fiocruz, Rio de Janeiro 21040-900, Brazil

**Keywords:** personalized medicine, siRNA delivery, biopharmaceutical company, performance of siRNA in clinical trials

## Abstract

The depth and versatility of siRNA technologies enable their use in disease targets that are undruggable by small molecules or that seek to achieve a refined turn-off of the genes for any therapeutic area. Major extracellular barriers are enzymatic degradation of siRNAs by serum endonucleases and RNAases, renal clearance of the siRNA delivery system, the impermeability of biological membranes for siRNA, activation of the immune system, plasma protein sequestration, and capillary endothelium crossing. To overcome the intrinsic difficulties of the use of siRNA molecules, therapeutic applications require nanometric delivery carriers aiming to protect double-strands and deliver molecules to target cells. This review discusses the history of siRNAs, siRNA design, and delivery strategies, with a focus on progress made regarding siRNA molecules in clinical trials and how siRNA has become a valuable asset for biopharmaceutical companies.

## 1. History of Interference RNA: Discovery and Mechanism of Action

### 1.1. Discovery

Modulation of gene expression via endogenous RNA interference pathways in mammalian cells is a powerful natural cellular process that promotes degradation of messenger RNA (mRNA) through the presence of interference RNA (miRNA, siRNA, RNAi). The natural functions of siRNA and its related processes act as a form of protection of the genome against invasion by foreign nucleic acids such as viruses and transposons [1,2].

The discovery of the siRNA complex revolutionized the performance of cell signaling pathways and played an important role in gene regulation. In 1998, in an unprecedented study, Fire and Mello, winners of the Nobel Prize, described the effective inhibition mechanisms of a gene in the C.elegans roundworm. In 1999, Tomari and Zamore discovered siRNA in plants acting similarly in the sequence-dependent endonucleolytic cleavage of mRNAs. These discoveries transformed our understanding of gene regulation by revealing that those responsible for the silencing were double-stranded RNAs that acted by degrading mRNA, revolutionizing studies in the areas of genetics and therapeutic applications [3,4,5].

### 1.2. Mechanism of Action

The basis for the development of applications using siRNA was elucidated in 2001 by Elbashir et al., who succeeded in silencing using these molecules and determined the principles of the structure and mechanics of siRNA. Those authors also demonstrated that the mechanism of action of these molecules as drugs was the inhibition of the expression of target genes by siRNA [4]. Since then, several new therapies, specific to different diseases, have emerged.

**Figure 1:**
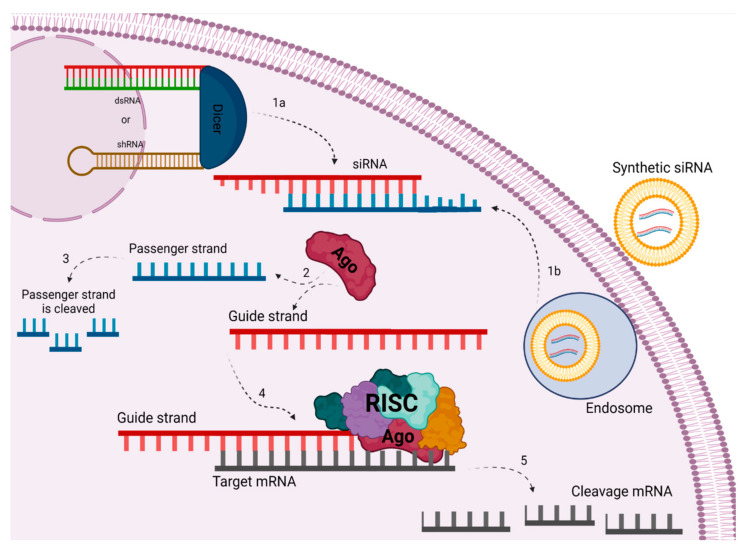
Gene silencing mechanism through siRNA in eukaryotic cells by different pathways: Through the endogenous pathway, long precursors, i.e., dsRNA or shRNA, are cleaved by the enzyme DICER into mature siRNA (1a). Via the exogenous pathway, the synthetic siRNA will enter a vesicle through the endosome and will be released into the cytoplasm (1b). Strand separation is done by the AGO protein (2), where the passenger strand will be cleaved (3) and the guide strand will be selected by the RISC complex to follow as a template for alignment with the mRNA (4). This will then be cleaved, thereby silencing the target gene (5). Created with biorender.

siRNA is just one point in a network known as RNA silencing, which has different pathways triggered by “small RNAs”, known as small interfering RNAs (siRNAs) and micro RNAs (miRNAs). As a component of this siRNA complex, small interfering RNAs (siRNAs; small RNAs of about 21 to 23 nucleotides and approximately 7–8 nm in length and 2 to 3 nm in diameter) present themselves as double-stranded RNA molecules consisting of a sequence which is complementary to the target mRNA with the potential to “silence” the expression of specific genes without producing an interferon response. These small RNAs guide the RNA-induced silencing effector complex (RISC) which shares a set of proteins, often called “siRNA machinery”, that play a role in the mRNA silencing process [5]. The performance of the interfering RNA can be divided into endogenous and exogenous processes (Figure 1). The endogenous siRNA process begins in the cytoplasm with the Dicer endoribonuclease, which degrades longer double-stranded RNA (dsRNA) or short hairpin RNA (shRNA) producing small interfering RNAs (siRNAs), which will be incorporated into the RISC complex. This mature siRNA is between 21–23 bases in length and typically features 2 bases phosphorylated at the 3’ end of each strand. Processing of the mature siRNA molecule is initiated by the RISC complex, separating the sense and antisense strands. The sense strand will be released by the complex, with the antisense strand remaining and serving as a guide to conduct the alignment with the target mRNA sequence. After alignment, Ago-2 endonucleases mediate cleavage of the target sequence. The use of siRNA for therapeutic purposes, for example, characterizes the exogenous process of this pathway. This ignores the initial step mediated by Dicer, where the processing of the longest double strand would occur, since the molecules are already introduced in mature form, prepared artificially and administered directly. In this pathway, the process in the RISC complex starts with the siRNA strands being unwound by the Ago2 protein and the degradation of the sense strand. The antisense strand, or guide strand, pairs with the complementary mRNA, leading to degradation and silencing of the target gene. This action may allow transient gene silencing by further degrading target mRNA for 3–7 days in rapidly dividing cells, or even for several weeks in slowly dividing cells, as the RISC complex remains activated with the strand antisense [4,6].

## 2. siRNA Design and Delivery to Human Cells

As the use of siRNAs was becoming more widespread, a crucial question was raised: why were some siRNA sequences effective in silencing the intended target and others not? in 2003, Khvorova et al. used computerized methods to compare sequences and thermodynamic profiles of hundreds of siRNAs and demonstrated that the structure and sequence of siRNA determine which strand will enter the RISC complex and participate in the RNA silencing pathway and which will be excluded. Therefore, some siRNAs appear inactive in vivo because the wrong strand followed the alignment path in the siRNA pathway. Given this, the strategy for designing siRNAs must be carefully reviewed, being essential to synthesize an antisense strand that optimizes selectivity and potency, and that does not bind to partially homologous mRNA sequences different from the target. The thermodynamic stability of the ends determines selection by the RISC complex; the asymmetry of one strand relative to another is an important characteristic that aids in the preference of this selection. The size difference can be achieved by modifying the ends of the strands, a very common strategy being the methylation of the 5’ end of the passenger strand. The 5’ end with greater stability is selected by RISC for the mRNA alignment process; these siRNAs are called “functionally asymmetric” [5,6,7].

The siRNA functionality must also consider the accessibility of the mRNA target site, as the folding of this site can block access and affect the siRNA potency. The choice of target sequences with greater accessibility should be considered, and the use of secondary structure prediction algorithms can help. Likewise, the self-folding of siRNA can also prevent recognition of the target site, and thus, inverted repetitions in the construction of sequences should be avoided [8,9].

The activity of siRNA can also be affected by internal double-stranded stability. An analysis of siRNA libraries showed that the most active sequences contained 36–52% guanine plus cytosine. It also demonstrated that the loading of the RISC complex can be affected by sequences with higher CG content, while lesser GC contents can affect the hybridization between the guide strand and the mRNA, weakening the siRNA activity [10].

Challenges beyond the construction of the siRNA sequence permeate the functionalization of this molecule. The easy degradation of siRNA in blood by nucleases and its subsequent elimination by glomerular filtration makes its half-life very short, preventing its action. Furthermore, its high hydrophilicity, anionic charge, and high molecular weight (~13 kDa) make diffusion through cell membranes difficult and create incompatibility with its objective [6]. Another difficulty of diffusing siRNA through the membrane occurs due to the deprotonation of the RNA phosphate groups at physiological pH. Given this, a vast number of delivery materials have been developed to minimize this vulnerability and improve the effectiveness of systemically administered siRNAs.

Another strategy to optimize the efficiency of siRNA and inhibit possible side effects is RNA chemical modifications, which have been proposed by some authors and companies. The main changes are in the phosphate backbone, ribose, or base [11]. It has been suggested that modification to the siRNA phosphate backbone makes it more potent and confers a longer half-life of the duplex. Ribose modification increases the half-life in the serum, reducing immune activation, and offers enhanced resistance towards nucleases. In a similar manner, the base modification also enhanced resistance towards serum nucleases [12]. Another strategy has been to use polysaccharides, such as chitosan.

To overcome the intrinsic difficulties of the siRNA molecule, therapeutic applications require nanometric delivery carriers, called nanovectors or nanoparticles (NPs), aiming to protect the double-strand and deliver the molecule to its target cell. Delivery molecules have been the biggest challenge for the therapeutic application of these siRNA molecules, since they must be formulated with non-toxic, biocompatible, biodegradable, and non-immunostimulating properties [13]. The types of delivery used in siRNA-focused therapy are classified into lipidic and polymeric materials. Cationic lipids can efficiently deliver siRNA using cationic liposome/siRNA complexes (lipoplexes) [14,15]. Lipid particles have some advantages, such as the low immunogenicity and easy integration with the cell membrane. Lipid-based nanoparticles and exosomes are, perhaps, two of the most promising vectorization strategies for gene delivery, reflected by the number of proposed clinical trials using these nanovectors for cancer [16]. With respect to polymeric particles, we can highlight the nanoparticles based on cationic polymers, which are composed of amines that can be protonated and therefore interact with siRNAs and condense them to form polyplexes [17].

One of the main challenges in the development of therapies using RNA interference technology is related to the delivery of the siRNA to the target cells. This is due to several structural factors of siRNA molecules, such as their instability, non-targeted biodistribution and the activation of an unwanted immune response [18]. In recent years, with advances in research involving this technology, several strategies have been studied, aiming to overcome these challenges. One of these strategies is through chemical alterations, which can be performed directly to the nucleotides of the siRNA molecule [19]. Another promising strategy is the use of delivery systems, which can help to increase the effectiveness, specificity and safety of the treatment [19]. One type of nanoparticle widely used as a delivery system is liposomes, which, as observed in Patisiran, allow a high rate of siRNA encapsulation and have good pharmacokinetic characteristics [20]. As we can see in Table 1, the direct conjugation of siRNA to ligands, which can be carbohydrates, peptides or even antibodies [19], is very frequent as a delivery system strategy. One of the most commonly used conjugates is GalNAC, a sugar derived from galactose, capable of allowing the delivery of siRNA specifically to liver cells via ASGPR (asialoglycoprotein receptor), which makes this ligand an excellent delivery system strategy in this tissue [21].

The therapeutic potential of this method is wide; there are therapies based on siRNA in development for the treatment of several diseases such as viral infections [22], hereditary diseases [23], and cancers [24]. Regarding viral infections, with the SARS-CoV2 pandemic, some therapies were proposed using siRNA as a way to block Covid-19. Some articles suggested that RNA could be used as the treatment, either to control SARS-CoV2 infections or to contain the cytokine storm. Two main strategies can be used to block viral infection, silencing (i) viral proteins which are essential for the survival and replication of SARS-CoV-2, or (ii) host factors involved in cellular entry and trafficking of the virus. Early studies indicated the potential of the siRNA therapeutics for COVID-19, three companies created libraries of siRNA for inhaled formulations [25,26]. Another important advantage of RNA therapy is the long-lasting effect of siRNAs. This effect was shown in a recent clinical trial where inhibition of the PCSK9 gene decreased the level of LDL cholesterol even after six months from the treatment [27]. This long-lasting effect of siRNA is a benefit for patients for whom it may not be feasible to receive frequent treatments [28]. Researchers at the MD Anderson Cancer Center in Houston have used this approach in the treatment of prostate cancer.

Many nanoparticles are functionalized, which implies the use of some ligands to direct these nanoparticles to the target cell. After selecting the best option of formulation for delivery using, or not, functionalization and achieving successful integration with the cell membrane, it is necessary to overcome the last obstacle, which is endosomal escape. After the siRNA-nanoparticles interact with the cellular membrane, the nanoparticles are engulfed in early endosomal vesicles and then fuse with late endosomes that form endosome-lysosome fusion. The lysosome environment is extremely acidic, resulting in siRNA degradation. Therefore, the study of endosomal escape strategies could help in the optimization of therapeutic nanoparticles. In this sense, fusogenic molecules have been used in delivery systems to allow the release of content into the cytoplasm or lipids such as 1,2-dioleoyl-sn-glycero-3-phosphoethanolamine (DOPE) that destabilize the endosomal membrane. Another approach is the use of small molecules, peptides, and proteins which promote the rupture of the endosomal membrane to prevent siRNA entrapment [12,16]. The use of these particles is also an important point in the functionality of the siRNA molecule, as they can affect the loading of the RISC complex. A delivery particle must perform its function very well, having to dissociate from the siRNA inside the cell, allowing the activation and loading of the RISC. Delivery materials can help internalize the siRNA double strand, releasing it through a shape transition, such as the conversion from lamellar to hexagonal phase in acidic environments. Alternatively, the molecules can be conjugated directly to the ends of the siRNA strands, usually to 3’ or 5’ ends of the passenger strand or up to the 3’ end of the antisense strand, as the 5’ end of this guide strand is essential for loading and activating the RISC complex [29].

Regardless of the type of nanoformulation, the efficiency of siRNA silencing is directly related to its physicochemical properties, with size being a critical characteristic for the performance of the nanocomplex for delivery. Among different types of nanoformulations, lipid nanoparticles are currently the most widely used in therapeutic applications using siRNA [19]. Within this context, nanoformulation techniques promise the delivery of preparations of varying sizes, encapsulated or complexed, through different systems, which are chosen depending on the physicochemical properties of the formulations and the materials to be encapsulated. Some of the most common preparation methods are lipid film hydration, microemulsification, sonication, extrusion and reversed-phase evaporation [2]. However, some of these methods generate a high rate of variability between batches, making large-scale production with reproducibility unfeasible [19].

In this context, the microfluidics technique, which was established by Jahn and collaborators in the preparation of liposomes in 2004, stands out, as it allows the controlled preparation of nanoparticles in seconds, with high precision, sample homogeneity and batch reproducibility, in addition to reducing the number of steps and achieving greater control and confidence in the process, including in both bench and large-scale use [3,4,5].

Microfluidic mixing is performed using a geometrically favorable device, where the lipids in a solvent are mixed with an aqueous buffer system through micromixers that increase the proportion of contact surface area between the liquids with a decrease in volume, which significantly increases the effectiveness of the mixture. These properties directly influence the nanoformulation parameters, such as size and polydispersity index (PDI), which are critical for its effectiveness. The microfluidic technique allows large-scale validation and reliable synthesis of nanoformulations with siRNA, and is currently considered the most promising platform [7,19].

Although the main objective of using siRNA is focused on its specific therapeutic effect on human target cells, the systemic effects that siRNAs and delivery molecules can induce must be taken into account, such as toxicity, off-target effects, and immunogenicity. Several factors can affect siRNA potency through degradation, elimination, and cellular uptake of delivery systems

## 3. siRNA Therapy in Clinical Studies and the Progress of Biopharmaceutical Companies

**Figure 2:**
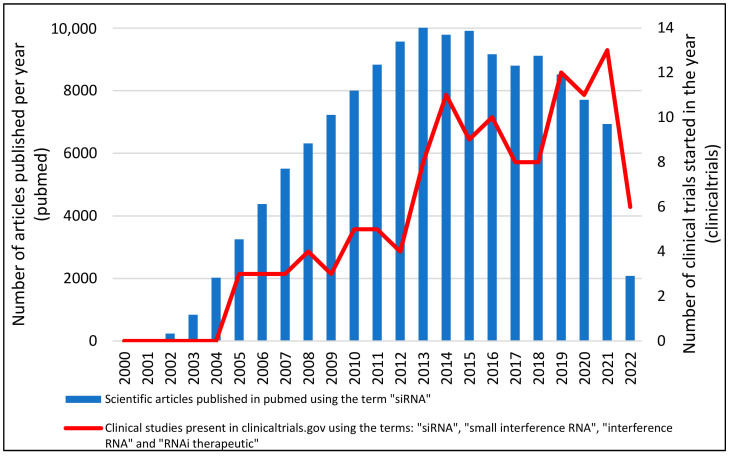
Number of articles and Clinical Studies about siRNA between 2000 and 2021. Data was obtained from the number of clinical trials present in clinicaltrials.com (accessed on 7 March 2022) using the terms: “siRNA”, “small interference RNA”, “interference RNA” and “therapeutic siRNA”. After data collection, duplicate data were excluded.

The first clinical study using siRNA was initiated in 2004, intending, in addition to determining the role of IL-10 in the pathogenesis of pre-eclampsia, to develop a treatment for pre-eclampsia using an IL-10-targeted siRNA (NCT00154934). At the end of that same year, another clinical study involving siRNA was started. In that study, siRNA-027 (AGN211745), a siRNA targeting vascular endothelial growth factor receptor-1, was developed for the treatment of Age-Related Macular Degeneration (AMD). In this study, the researchers evaluated the safety, toxicity, and anatomical and functional changes presented in patients treated with only one dose of the siRNA under study (NCT00363714) [30]. Following these original studies, the number of clinical studies using siRNA technology has been growing at an accelerated rate, reaching the mark of 15 clinical studies initiated in the year 2021 (Figure 2). In the 2000s, according to clinicaltrials.gov, 18 clinical trials were started involving the term siRNA. In the 2010s this number rose to 76 and in these first two years of the 2020s, 25 clinical studies were started. These data demonstrate how promising this new technology is.

Studies have already identified siRNA therapies which will soon be commercialized as biopharmaceuticals [31]; however, it was two decades after the discovery of siRNA that the first siRNA based therapy was approved by the Food and Drug Administration (FDA) and the European Commission (EC) for commercial use. The significant increase in clinical trials initiated in recent years, mainly in 2021, is believed to be due to the approval by the Food and Drugs Administration (FDA) of the first therapeutic siRNA in 2018, i.e., Patisiran (ONPATTRO™). The drug from the company Alnylam Pharmaceuticals was approved for the treatment of transthyretin amyloidosis (ATTR), a rare inherited disease caused by genetic mutations that cause a malformation of the protein transthyretin (TR), which affects people progressively and is usually fatal [32]. This drug is composed of a small double-stranded interfering ribonucleic acid (siRNA) which targets the mRNA that translates to mutant TTR, encapsulated in a liposome. The liposome is a lipid nanoparticle capable of delivering siRNA to hepatocytes, where it will specifically recognize and degrade the mutant transthyretin messenger RNA, decreasing the production of this protein and consequently reducing serum levels and deposits of this protein in tissues [33]. To date, 16 clinical studies have been registered using this drug, 5 of which are phase 3 studies.

After the approval of the first therapy using siRNA, three other agents were approved using this technology (Givosiran, Lumisiran and Inclisiran) and many others are already in advanced stages of development with phase three clinical studies having already been initiated. The second treatment to be approved was Givosiran (Givlaari™; Alnylam Pharmaceuticals, Massachusetts, EUA), also developed by Alnylam Pharmaceuticals. This drug was approved in late 2019 by the FDA for the treatment of adults with acute hepatic porphyria (AHP), a group of rare genetic disorders caused by mutations that generate dysfunctions in specific heme biosynthesis enzymes, resulting in the abnormal accumulation of intermediaries of this pathway [34,35]. In 2020, this drug was also approved by the European Union (EU) for the treatment of adults and adolescents with AHP [35]. Givosiran is a therapeutic siRNA that acts by cleaving the mRNA of aminolevulinate synthase 1 (ALAS1), one of the enzymes involved in the biosynthesis of heme and which lowers blood levels of aminolevulinic acid and porphobilinogen, neurotoxic intermediates that are associated with symptoms in patients [36]. To allow effective delivery of therapy to hepatocytes (affected tissue), siRNA is covalently linked to the monosaccharide N-acetylgalactosamine (GalNAc) [35,37].

The third therapy using siRNA technology to be approved was Lumasiran (Oxlumo™; Alnylam Pharmaceuticals, Massachusetts, EUA), which is composed of a siRNA that is capable of silencing the expression of glycolate oxidase, an enzyme capable of synthesizing glyoxylate. With this treatment, there is a reduction in available glyoxylate levels and, consequently, in oxalate production, reducing urinary and plasma oxalate levels [34,38,39]. This therapy was developed by Alnylam Pharmaceuticals and was approved by the EU and FDA in November 2020 for the treatment of primary hyperoxaluria type 1 (PH1) in adult and pediatric patients. This is a rare autosomal recessive metabolic disease caused by a deficiency of the hepatic enzyme alanine-glyoxylate aminotransferase (AGT), which generates an accumulation of oxalate in the affected tissues [38,39]. It is noteworthy that this therapy uses the chemical enhanced stabilization platform (ESC) combined with N-acetylgalactosamine (GalNAc) as the siRNA delivery platform [34]. There are currently 7 clinical studies registered with this therapy.

Inclisiran (Leqvio™; Novartis, Basel, Switzerland) was the fourth and newest approved therapeutic siRNA. In this drug, siRNA is conjugated to GalNAc residues and aims at silencing the hepatocyte proprotein convertase subtilsin/kexin type 9 (PCSK9) through the degradation of its mRNA. This protein participates in the degradation of low-density lipoprotein (LDL) receptors, which consequently regulates plasma cholesterol levels [40,41,42]. The therapy developed by Novartis received, in December 2020, its first approval by the European Medicines Agency (EMA) for the treatment of hypercholesterolemia or mixed dyslipidemia in adult patients [40]. In December 2019, Inclisiran had already been submitted for FDA approval through a new drug application (NDA) for the treatment of patients with atherosclerotic cardiovascular disease (ASCVD) and hypercholesterolemia, however, by the end of 2020, the application was responded with a full response letter (CRL) due to unresolved conditions related to inspection of the manufacturing facility. In July 2021, Novartis resubmitted an NDA and is awaiting review by the FDA for approval [40,43].

In addition to the approved siRNAs, several clinical trials have been registered at clinicaltrials.com (accessed on 7 March 2022) using siRNA as therapies for various diseases. In the Table 1, we summarize clinical trials using siRNAs as a therapy that are in advanced stages of development or that have been approved for the market.

As can be seen in Table 1, Alnylam Pharmaceuticals is a pioneer company that leads in the ranking of companies involved in the development of therapies using siRNA. This company is responsible for three of the four siRNA approved to date. Furthermore, it is worth noting that most clinical trials are related to metabolic and rare diseases. Great efforts have been put into bringing therapeutic siRNA to the market. The biopharmaceutical companies that have been investing in siRNA development for the past 25 years are presented in this paper and their products are listed in Table 2. It is well established that nucleic-acid-based biopharmaceuticals are entering the market with the help of new biopharmaceutical companies. Since 2003, companies like Alnylam Pharmaceutical and siRNA Therapeutics have undertaken research that could result in clinical applications of siRNA. In 2006, the big pharma industry began to forge partnerships with these small companies to gain access to this technology. Despite initial enthusiasm, the application of siRNA in the clinic did not take off at the time. The lack of companies specializing in delivery system and the lack of stability of siRNA were cited as bottlenecks at the time. Some companies persisted, and in 2007, data from clinical trials finally began to justify the persistence. In 2010, a study demonstrated gene silencing in humans, and in 2013, Alnylam published the first clinical trial data of Patisiran. To this day, several other biotech companies also have siRNA candidate drugs in their pipelines. Here, we present a list of the main companies involved in siRNA technology. Many of these companies are a consequence of partnerships, and therefore, the complete mapping of them becomes a difficult task. Most siRNA-based companies are targeting products for central nervous system diseases, primarily Huntington’s disease and Alzheimer’s disease; Alnylan and Dicerna are examples of this.

## 4. Pros and Cons for the Broader Use of siRNAs into Clinics

Since Elbashir and colleages [44] and Caplen and colleages [45] reported that dsRNAs could induce silencing in mammalian cells, siRNA molecules have become an important tool in biological research, as they allow easy and rapid gene expression inhibition in a sophisticated and delicate way, thus becoming key for precision medicine. After 20 years of investigation, a large amount of data regarding siRNA has been compiled, helping these molecules to be recognized as an attractive modality for precision medicine therapies as a new class of drugs.

**Figure 3:**
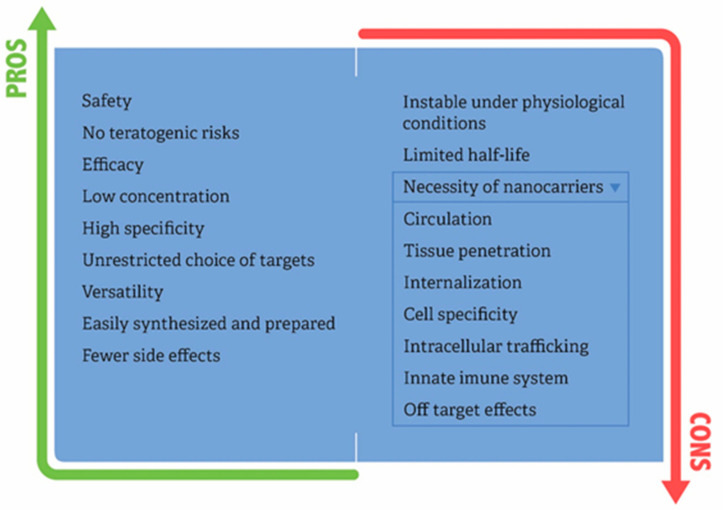
Pros and cons for the broader use of siRNAs into clinics.

The advantages of siRNA over other therapeutic modalities include a list of features (Figure 3).

(1) siRNA has a high degree of safety, as it acts on the post-translational stage of gene expression, and therefore, does not interact with DNA, thereby avoiding mutation and teratogenic risks common to gene therapy. Based on clinical studies, siRNA therapeutics were relatively well tolerated, and cytokine release and infusion-related adverse events were manageable with supportive treatments, such as dexamethasone, acetaminophen, diphenhydramine, and ranitidine [46].
(2) siRNA is highly efficacious in a precision therapeutic arsenal, once genes can be silenced by over 90%. Based on this feature, siRNA has innate advantages in comparison with small molecule therapeutics and monoclonal antibodies, because siRNA executes its function by complete Watson–Crick base pairing with mRNA, whereas small molecule and monoclonal antibody drugs need to recognize the 3D spatial conformation of proteins.
(3) siRNA can cause dramatic suppression of gene expression in a single cell with just a few copies.
(4) It has high specificity; in some cases, a single point mutation can abrogate silencing effect [47]. To take advantage of the sequence specificity of siRNA, a prerequisite to achieving allele-specific gene silencing is to identify the most significant difference between two alleles, which may be as little as a single nucleotide change stemming from mutation or polymorphism [48].
(5) siRNA presents versatility because interfering RNA can be designed against virtually any gene.
(6) Ease of synthesis, with lower production costs compared to protein or antibodies, and no need for a cellular expression system and complex protein purification.

Additionally, the pros of siRNA used in therapy go beyond cytoplasmatic mRNA silencing. The approval of ONPATTRO brings new hope to patients with hATTR, but also for a number of genes or signaling pathways that are ‘undruggable’ by classical small molecules. Furthermore, siRNA could be used in difficult-to-treat diseases, such as cancer, as well as metabolic, autoimmune and rare diseases. In this scenario, siRNA definitively opens up new horizons for drug development. More specifically, these molecules have been paving the way to creating ‘programmable’ drugs that can specifically turn off genes and at the desired time.

Several computational design tools, protocols, and validated commercially available molecules have been helpful to scientists for sequence choice and siRNA design. The efficiency of siRNA molecules depends on different factors, including target availability, secondary structures of mRNA, the position of matching, and intrinsic characteristics of siRNA and mRNA [10]. Eight characteristics associated with siRNA functionality were identified by Reynolds et al. [49]: low G/C content (36–52%), a bias towards low internal stability at the sense strand 3’-terminus, lack of inverted repeats and sense strand base preferences (positions 3, 10, 13 and 19). It has been proposed that higher GC content may inhibit RISC loading and release of the cleaved sense strand, while lower GC content may inhibit activity by weakening hybridization between the guide strand and mRNA [48]. To circumvent these issues various solutions have been available and reviewed by Fakhr et al. [50] in terms of applying good designing methods and available software. Sequence selection has an overpowering effect on strand selectivity (safety), on-target potency, and off-target effect [48].

The technological advances in the -omics approaches have revolutionized the development of siRNAs drugs, which increased the maturity and sophistication of gene selection. Key lessons learned include the benefits of preclinical and clinical tests, in particular related to a careful optimization of formulation stability and quality control in future clinical trials. Many successful strategies have been developed by rational design using high throughput screenings, especially based on -omics data-integration approaches and genome-wide siRNA screening [51,52]. Furthermore, it is important to highlight that siRNA-based methods are helpful in the context of understanding the complex basis of a number of diseases such as cancer, infection, HIV [53], neurodegenerative and respiratory diseases. Since 2001, thousands of cancer-based studies have reported the employment of siRNA-based methods to study mammalian gene function, leading to the discovery of many genes with no previous links to cancer biology, for example, and enhanced understanding of the functions of established oncogene and tumor suppressors [54]. siRNAs have been extensively used in cell-based studies for pathways dissection and to knockdown the expression of genes involved in a range of cellular processes, including endocytosis, signal transduction, metabolism, apoptosis, necrosis, autophagy, cell cycle, and genomic catastrophe events.

Davis et al. [55] conducted the first-in-human phase I clinical trial (clinical trial registration number NCT00689065) involving the systemic administration of siRNA to patients with melanoma cancer using a targeted, nanoparticle delivery system. Thirteen patients with melanoma cancer refractory to standard-of-care therapies are administered doses of targeted, nanoparticles on days 1, 3, 8, and 10 of a 21-day cycle via a 30-min i.v. infusion. The nanoparticles consist of a synthetic delivery system containing polymer, a human transferrin protein (TF) targeting ligand, and siRNA designed to reduce the expression of the M2 subunit of ribonucleotide reductase (RRM2). This was the first clinical trial to systemically deliver siRNA with a targeted delivery system and to treat patients with solid cancer. These data provide evidence of inducing a siRNA mechanism of action in a human from the delivered siRNA. Tumour biopsies from melanoma patients obtained after treatment show the presence of intracellularly localized nanoparticles in amounts that correlate with dose levels of the nanoparticles administered. Furthermore, a reduction was observed in both the specific RRM2 messenger RNA and the RRM2 protein levels when compared to pre-dosing tissue. These data pave the way for the administration of siRNA systemically to humans.

The other side of the coin regarding the use of siRNAs in clinical practice is related to their chemical and biological properties. siRNA molecules are small in size (molecular weight ~13 kDa), hydrophilic, and negatively charged, and they cannot diffuse across biological membranes alone. For siRNA therapies to be applied in clinical practice, it is first necessary to determine how to deliver the siRNA safely to target organs. Two administration routes used: localized and systematic administration (i.v.). Localized siRNA administration has fewer barriers than systemic delivery. In the localized delivery of siRNA, therapy is applied directly to the target organ or tissues, offering high bioavailability at the target site, and avoiding several biological barriers that are faced by the systemic administration. It is possible to apply local or topical siRNA delivery in eyes, mucous membranes, localized tumors, and skin. Grzelinski et al. [56] showed that orthotopic mouse glioblastoma model with U87 cells growing intracranially treated with polyethylenimine-complexed pleiotrophin (PTN) siRNAs injection into the CNS exerts antitumoral effects that account for 40% of tumor volume reduction. Lung infections or diseases can be treated with local siRNA delivery by inhalation of siRNA through intranasal routes which permits direct contact with epithelial cells of the lungs [57]. However, systemic administration is the most sought after due to the minimization of side effects and also because it is more comprehensive in terms of accessibility to the diseased tissue. Based on the intrinsic features of siRNAs, it is mandatory that siRNA needs to be packaged in a suitable nanocarrier or chemical modifications are necessary. The pharmacodynamics and pharmacokinetics of siRNA delivery systems are dependent not only upon the siRNA therapeutic but also upon the biomaterials included in the delivery vehicle. Ways are needed to shield the siRNA from degradation in the bloodstream, prevent it from being filtered out by the kidneys, to allow it to exit blood vessels, spread through target tissues, and enter specific cells in order to turn-off the target gene. siRNA therapy faces several extracellular and intracellular barriers for clinical translation. Detailed limitation steps for the use of siRNAs and delivery systems into clinics are presented (Figure 3):

(1) Blood circulation: siRNA is not stable under physiological conditions because it is susceptible to serum nuclease-catalyzed degradation. The phosphodiester bond of siRNA is vulnerable to RNases and phosphatases. Once it is intravenously administered, endonucleases or exonucleases throughout the body will degrade siRNA, thus preventing the accumulation of intact therapeutic siRNA in the target tissue. The half-life of naked or unmodified siRNA in serum ranges from several minutes to 1hour. Consequently, siRNAs present poor pharmacokinetic behavior. Regarding chemical modifications, the most common strategy involves modification of the ribose 2′-OH group, as this functional group is critical to the mechanism of many serum RNAses [10]. Among the most effective backbone modifications for serum stability improvement is the substitution of the ribose 2′ hydroxyl with 2′-fluorine or 2′-methoxy groups. However, siRNA modification alone may not be enough to achieve the therapeutic activity. In this way, physical encapsulation of siRNA promotes stability to proper therapeutic activity. Concerning delivery systems, non-specific interactions with serum proteins result in nanocarrier degradation, dissociation, or aggregation. In addition to degradation by circulating nuclease, another barrier to in vivo delivery of siRNA is the uptake by the reticuloendothelial system. The reticuloendothelial system is composed of phagocytic cells, including circulating monocytes and tissue macrophages, which act to clear foreign pathogens and to remove cellular debris and apoptotic cells. Tissue macrophages, called Kupffer cells, are most abundant in the liver and spleen, tissues that receive high blood flow. Among nanocarrier options, PEGylated nanoparticles were highly efficient in delivering siRNA to the tumor with low liver uptake by evasion of the reticuloendothelial system [58].
(2) Tissue carriage and internalization: The major concern about siRNA, besides degradation, is the transport from the bloodstream to the desired tissue. siRNA is ~7–8 nm in length and 2–3 nm in diameter. Therefore, these molecules are too large to cross cell membranes but small enough to be freely cleared by glomeruli, as molecules with a size smaller than 8 nm are easily filtered by the renal system. Hence, once siRNAs leave the bloodstream, they will accumulate in the bladder and be excreted from the body quickly. Additionally, chemical properties of siRNAs also interfere with the ability to cross the cell membrane, more specifically their relatively high molecular weight (~13–16 kD) and negative charge. These observations reinforce the notion that siRNAs should be encapsulated in order to overcome clearance, degradation and enable cell penetration.
(3) Extracellular stability: In comparison with the pH values in the blood and healthy tissues (pH 7.4), the pH values in the tumor microenvironment, for example, have been found to range from 6.0 to 7.2 [59]. These differences in the pH, together with enzymes or ions of the tissue microenvironment could injure the nanocarrier, causing dissociation and releasing of the cargo before cellular entry.
(4) Cell specificity: The rational is to coat the nanocarrier with a receptor, specific for the cell type to be targeted and, therefore, taken up by the targeted cells only. Presently, nanoparticles are frequently used for specific siRNA delivery in clinical trials due to the stringent specificity possessed by them. Davis et al. [55] conducted the first-in-human phase I clinical trial involving the systemic administration of siRNA to patients with solid cancers using a targeted, nanoparticle delivery system. The authors developed a nanoparticle coated with a human transferrin protein targeting ligand, displayed on the exterior of the nanoparticle, to engage Tf receptors on the surface of the cancer melanoma cells. The same strategy was used in Patisiran therapy, to target receptors that transport low-density lipoproteins (LDLs) into the cell.
(5) Innate immune system: High levels of siRNA have been known to result in the activation of innate immune responses, immunostimulation, immunosuppression, and the production of cytokines both in vitro and in vivo [60]. Toll-like receptors (TLRs) 3, 7, and 8 are involved in the recognition of siRNA. siRNA has been found to activate TLR3, signaling in a sequence-independent manner. However, TLR7 and TLR8 mediate the recognition of siRNA in a sequence dependent manner that could be activated by sense or antisense strand. Thus, the importance of the sequence in siRNA-mediated immune stimulation requires more investigation [61].
(6) Intracellular trafficking: Although it is commonly accepted that siRNA enters into cells via endocytosis, this broad statement masks the complexities inherent to the multiple pathways of internalization and subsequent intracellular trafficking, and this complexity is based on the chemical composition of the nanocarrier [62]. Subcellular membrane bound compartments include early and recycling endosomes, late endosomes, lysosomes, the Golgi apparatus, and the endoplasmic reticulum. Trafficking is not a random process, but rather, it is a carefully orchestrated pathway that allows the cell to transport endogenous and exogenous materials to the most appropriate place. The main point of concern regarding siRNA and intracellular trafficking is that siRNA needs to escape from vesicles to reach the cytoplasm, in order to find the siRNA machinery and avoid degradation induced by low pH into the vesicles.
(7) Off-target: An siRNA duplex may target more than one mRNA molecules, due to sequence homologies. It is now widely observed that most siRNAs can tolerate one mismatch to the mRNA target and, at the same time, retain good silencing capacity [62,63,64]. Off-target effects of siRNA lead to unanticipated phenotypes that complicate the interpretation of the therapeutic benefits. RISCs can potentially downregulate any mRNAs with perfect base-pairing complementarity to the guide-strand seed region. This limitation could be circumvented in the siRNA design step. It is extremely important to use, for example, the NCBI BLAST tool to avoid matching to other non-desired sequences. Another critical issue with siRNA therapeutic development in cancer is an off-target accumulation of the delivery vehicle and its cargo, which depends on the chemical material used to produce it.

Thus, there is a need to optimize siRNA delivery systems to facilitate internalization into cells, the release of the drug from the delivery system and endosomes, and delivery into the cytoplasmic compartment of cells, further minimizing off-target delivery and immune activation. There are significant challenges that need to be solved before siRNA can be widely applied in the treatment of other genetic diseases like cancer.

## 5. Immune System Activation and Escape by siRNA

### 5.1. Immune Activation

**Figure 4:**
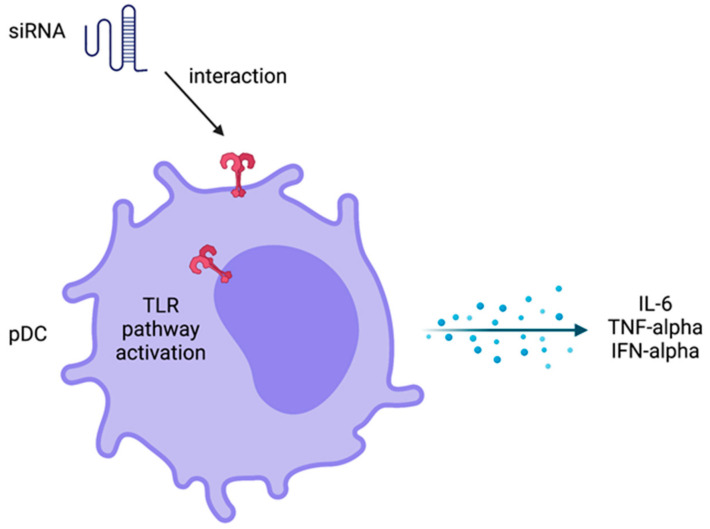
Innate immune response activation by siRNA. There is an interaction among siRNA and plasmacytoid dendritic cells (pDC) upon systemic delivery to induce the cell activation and cytokines production by toll-like receptor (TLR) signaling pathway. Created with biorender.

To design a delivery system, nanostructures need to be able to hide from the immune system. A successful therapy using siRNA design involves balanced immune activation. It is very important not to induce an exacerbated immune response, causing side effects, but also to trigger an effective immune response to increase the therapeutic effect. It has been shown that innate immune response can be activated by siRNA therapies, using systemic administration, promoting an increase of inflammatory cytokines, such as TNF-alpha, IL-6, and interferons, mainly IFN-alpha (Figure 4). Plasmacytoid dendritic cells can produce high levels of IFN-alpha when induced by siRNA in peripheral mononuclear cells from human blood using in vitro assays [65,66].

Another important point in the innate immune system that needs to be better explored when using siRNA therapy is the regulation of the toll-like receptor (TLR) pathway. TLR pathway alterations are the most common non-specific effects that may limit the use of siRNA therapy. With this in mind, changes of this pathway and their consequences should be investigated in non-clinical studies as well as in clinical trials [65,66].

T cells are another potential target for siRNA therapies. Their use has been considered an attractive and challenging approach, in which silencing of gene expression has two sides: [1] interference of the signaling pathway for T cell activation, and [2] T cell suppression that could regulate the T cell functionality for different purposes, such as cancer, inflammatory diseases, infections, etc. [67,68]. Chimeric antigen receptor (CAR) T cell therapy has been used in cancer treatment for leukemias and lymphomas. This therapy is already in use with engineered T cells, which can induce a change of the T cell functionality to permit the immune system fight against cancer. Simon et al., 2018, showed that siRNA could improve the functionality of CAR-T cell immunotherapy against cancer [69].

The challenges of the siRNA as a therapy to induce a good and effective immune response should be considered with caution. Additional and extensive studies are necessary, though it appears to be a promising technology to provide, in the near future, treatment for several diseases.

### 5.2. Immune Evasion Strategies

One of the main issues in the field of siRNA is potential nonspecific immunostimulatory effects. In this regard, target tissues and vehicles must be evaluated to determine the best delivery system. The choice of compartmentalized targets with direct access improves the potential of siRNA technology, while systemic therapies face a number of barriers [70]. Systemic siRNA administration enhances the risk of premature drug clearance by physiologic pathways such as kidney filtration, aggregation with serum proteins, and enzymatic degradation by endogenous nucleases, but also by an immunologic mechanism: the uptake by phagocytic cells [71].

Phagocytosis serves as a significant immunological barrier, not only in the bloodstream but also in the extracellular matrix of tissues. Unfortunately, phagocytes are also highly efficient at removing certain therapeutic nanocomplexes and macromolecules from the body [71]. The nanoparticles interact massively with the surrounding physiological environments, including plasma proteins, upon administration into the bloodstream. Consequently, they may be rapidly cleared [72].

**Figure 5:**
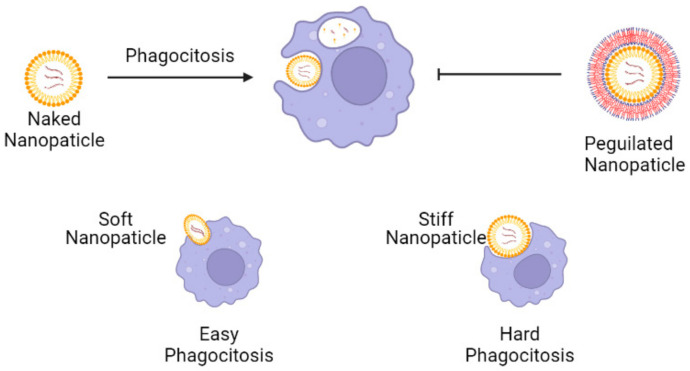
Nanoparticles containing siRNA and the strategies to evade phagocytosis. Nanoparticles coated with non-ionic hydrophilic polymers, as well as PEG (PEGylated nanoparticles) can evade phagocytic cells given the diminished adsorption of opsonin. Soft nanoparticles are more likely to be engulfed, given the ability of cells to deform nano capsules. Created with biorender.

In the phagocytic opsonization process, it was observed that hydrophobic nanoparticles were engulfed or evaded within the cytosol at higher concentrations as compared to hydrophilic nanoparticles, due to the more significant adsorption of opsonin, a molecule that enhances the phagocytic process, on their surface [73]. Thus, one traditional strategy to avoid phagocytosis is grafting a stealth coating layer onto the surface of nanoparticles using non-ionic hydrophilic polymers, such as Polyethylene Glycol (PEG), as siRNA vehicles [74,75]. Recently, Hui and colleagues conducted an in vitro study demonstrating that the elasticity of NPs significantly affects their cellular uptake, given the ability of cells to deform nanocapsules either considering clathrin-mediated endocytosis or clearance by phagocytosis [75]. Hence, PEG and balanced elasticity can be used as strategies to avoid nanoparticle clearance by phagocytosis (Figure 5).

## 6. Conclusions

The discovery of siRNA as a drug is considered one of the most significant therapeutic advances in pharmaceutical development, partly because this molecule can be used to target almost all disease-related genes. For years, researchers worked to overcome the various obstacles associated with the development of siRNA as a therapeutic modality and overcome the stagnation phase. However, in recent years, many advances in delivery and siRNA technologies have facilitated the development of siRNA drugs, and siRNA pharmaceuticals have already been approved for human use, while various others are in clinical trials and in the pipeline, and a number of companies and laboratories are specializing in this technology. This brings about the expectation that, although further research and development remain necessary to advance siRNA technologies, this therapeutic modality is now part of the arsenal for the treatment or long-term control of various untreatable or difficult to treat diseases.

## Figures and Tables

**Table 1:** Clinical studies of siRNA therapeutics.

Drug	Target	Delivery System	Administration	Disease	Company	Status	Phase	Study Start	NCT Number *
Patisiran (ONPATTRO ™) ALN-TTR02	TTR	Lipid Nanoparticle	IV infusion	TTR-mediated amyloidosis	Alnylam Pharmaceuticals	Completed	Phase 3	2013	NCT01960348
							Phase 3	2019	NCT03862807
						Active, not recruiting	Phase 3	2015	NCT02510261
							Phase 3	2019	NCT03759379
						Approved for marketing		2016	NCT02939820
						Active, not recruiting	Phase 4	2019	NCT04201418
							Phase 3	2019	NCT03997383
Givosiran (Givlaari ™) ALN-AS1	ALAS1	GalNAc conjugate	SC injection	AHP	Alnylam Pharmaceuticals	Completed	Phase 3	2017	NCT03338816
						Approved for marketing		2019	NCT04056481
						Recruiting		2021	NCT04883905
Lumasiran (Oxlumo ™) ALN-GO1	HAO1	GalNAc conjugate	SC injection	PH1	Alnylam Pharmaceuticals	Active, not recruiting	Phase 3	2018	NCT03681184
							Phase 3	2019	NCT03905694
							Phase 3	2020	NCT04152200
						Recruiting		2021	NCT04982393
						Approved for marketing			NCT04125472
Inclisiran (Leqvio ™) ALN-PCSsc	PCSK9	GalNAc conjugate	SC injection	ASCV and Elevated Cholesterol	Novartis	Completed	Phase 3	2017	NCT03399370
							Phase 3	2017	NCT03400800
						Enrolling by invitation	Phase 3	2019	NCT03814187
				Atherosclerotic Cardiovascular Disease		Recruiting	Phase 3	2018	NCT03705234
							Phase 3	2021	NCT04929249
							Phase 3	2021	NCT04807400
						Not yet recruiting	Phase 3	2021	NCT05030428
				Hypercholesterolemia		Recruiting	Phase 3	2021	NCT04652726
							Phase 3	2021	NCT04659863
							Phase 3	2021	NCT04765657
						Completed	Phase 3	2017	NCT03397121
						Active, not recruiting	Phase 3	2019	NCT03851705
						Not yet recruiting	Phase 3	2021	NCT05004675
								2021	NCT05118230
				ACS		Recruiting	Phase 3	2021	NCT04873934
Fitusiran ALN-AT3SC	AT	GalNAc conjugate	SC injection	Hemophilia	Genzyme	Active, not recruiting	Phase 3	2018	NCT03549871
						Completed	Phase 3	2018	NCT03417245
							Phase 3	2018	NCT03417102
						Recruiting	Phase 3	2019	NCT03754790
Vutrisiran ALN-TTRSC02	TTR	GalNAc conjugate	SC injection	ATTR	Alnylam Pharmaceuticals	Active, not recruiting	Phase 3	2019	NCT04153149
							Phase 3	2019	NCT03759379
Teprasiran QPI-1002	p53	None	IV injection	Delayed Graft Function	Quark Pharmaceuticals	Completed	Phase 3	2016	NCT02610296
				Cardiac Surgery		Terminated	Phase 3	2018	NCT03510897
QPI-1007	Caspase-2	None	Intravitreal	NAION	Quark Pharmaceuticals	Completed	Phase 2	2013	NCT01965106
Tivanisiran SYL1001	TRPV1	None	Ophthalmic solution	Dry Eye Disease	Sylentis, S.A.	Completed	Phase 3	2017	NCT03108664
						Recruiting	Phase 3	2021	NCT04819269
Nedosiran DCR-PHXC	HAO1	GalNAc conjugate	SC injection	Primary Hyperoxaluria	Dicerna Pharmaceuticals	Enrolling by invitation	Phase 3	2019	NCT04042402
Cemdisiran ALN-CC5	C5	GalNAc conjugate	SC injection	gMG	Alnylam Pharmaceuticals	Not yet recruiting	Phase 3	2021	NCT05070858
				Paroxysmal Nocturnal Hemoglobinuria			Phase 3	2022	NCT05133531
							Phase 3	2022	NCT05131204

* Clinical trial identification number registered at https://clinicaltrials.gov/ct2/ (accessed on 7 March 2022). Abbreviations: GalNAc, N-acetyl-d-galactosamine; HAO1, hydroxy acid oxidase 1; PCSK9, proprotein convertase subtilisin/kexin type 9; TRPV1, transient receptor potential cation channel subfamily V member 1; TTR—transthyretin; IV—intravenous; SC—subcutaneous; PH1—Primary Hyperoxaluria Type 1; ASCVD—atherosclerotic cardiovascular disease; ACS—Acute Coronary Syndrome; ATTR—Transthyretin Amyloidosis; NAION—Non Arteritic Anterior Ischemic Optic Neuropathy; gMG—Generalized Myasthenia Gravis; AHP—Acute Hepatic Porphyria.

**Table 2:** Biopharmaceutical companies involved in siRNA therapeutics development.

Pharmaceuticals Companies	Pathology
DTx pharma	Eye, neuromuscular, neurodegenerative, cardiovascular, immune, and oncology
Alnylam Pharmaceuticals, Inc.	Genetic medicines, cardio metabolic diseases, infectious diseases, ocular diseases, CNS (central nervous system diseases)
Phio Pharmaceuticals	Oncology
Silence Therapeutics	Rare diseases, cardiovascular disease
Aphios Corporation	Oncology, anti-infectives, CNS diseases such as Alzheimer’s Disease, Cognition, Depression, and Pain.
Dicerna Pharmaceuticals	Metabolic and cardio metabolic diseases, complement-mediated, hepatitis B
Avidity Biosciences	Muscular diseases
Sirnaomics, Inc	Oncology, fibrosis, antiviral
ARIZ Biopharma	Oncology
Atalanta Therapeutics	Neurodegenerative diseases
Entos pharmaceutics	Oncology, Age-related diseases
Arbutusus Biopharma	Products for HBV infection
Arrowhead	Products for cancer, viral infections, metabolic and rare diseases

## Data Availability

Data sharing not applicable.

## References

[1] Dong Y., Siegwart D.J., Anderson D.G. (2019). Strategies, design, and chemistry in siRNA delivery systems. Adv. Drug. Deliv. Rev..

[2] Agrawal N., Dasaradhi P.V.N., Mohmmed A., Malhotra P., Bhatnagar R.K., Mukherjee S.K. (2003). RNA interference: Biology, mechanism, and applications. Microbiol. Mol. Biol. Rev. MMBR.

[3] Fire A., Xu S., Montgomery M.K., Kostas S.A., Driver S.E., Mello C.C. (1998). Potent and specific genetic interference by double-stranded RNA in Caenorhabditis elegans. Nature.

[4] Dana H., Chalbatani G.M., Mahmoodzadeh H., Karimloo R., Rezaiean O., Moradzadeh A., Mehmandoost N., Moazzen F., Mazraeh A., Marmari V. (2017). Molecular Mechanisms and Biological Functions of siRNA. Int. J. Biomed. Sci. IJBS.

[5] Tomari Y., Zamore P.D. (2005). Perspective: Machines for RNAi. Genes Dev..

[6] Caillaud M., El Madani M., Massaad-Massade L. (2020). Small interfering RNA from the lab discovery to patients’ recovery. J. Control. Release.

[7] Zhang M.M., Bahal R., Rasmussen T.P., Manautou J.E., Zhong X. (2021). The growth of siRNA-based therapeutics: Updated clinical studies. Biochem. Pharmacol..

[8] Patzel V., Rutz S., Dietrich I., Köberle C., Scheffold A., Kaufmann S.H.E. (2005). Design of siRNAs producing unstructured guide-RNAs results in improved RNA interference efficiency. Nat. Biotechnol..

[9] Köberle C., Kaufmann S.H.E., Patzel V. (2006). Selecting effective siRNAs based on guide RNA structure. Nat. Protoc..

[10] Kanasty R.L., Whitehead K.A., Vegas A.J., Anderson D.G. (2012). Action and Reaction: The Biological Response to siRNA and Its Delivery Vehicles. Mol. Ther..

[11] Hu B., Zhong L., Weng Y., Peng L., Huang Y., Zhao Y., Liang X.J. (2020). Therapeutic siRNA: State of the art. Signal Transduct. Target. Ther..

[12] Sajid M.I., Moazzam M., Kato S., Yeseom Cho K., Tiwari R.K. (2020). Overcoming Barriers for siRNA Therapeutics: From Bench to Bedside. Pharmaceuticals.

[13] Ozpolat B., Sood A.K., Lopez-Berestein G. (2014). Liposomal siRNA nanocarriers for cancer therapy. Adv. Drug. Deliv. Rev..

[14] Zhang S., Zhao B., Jiang H., Wang B., Ma B. (2007). Cationic lipids and polymers mediated vectors for delivery of siRNA. J. Control. Release.

[15] Hattori Y., Nakamura M., Takeuchi N., Tamaki K., Shimizu S., Yoshiike Y., Taguchi M., Ohno H., Ozaki K.I., Onishi H. (2019). Effect of cationic lipid in cationic liposomes on siRNA delivery into the lung by intravenous injection of cationic lipoplex. J. Drug. Target.

[16] Mainini F., Eccles M.R. (2020). Lipid and Polymer-Based Nanoparticle siRNA Delivery Systems for Cancer Therapy. Molecules.

[17] Wang H., Zhang S., Lv J., Cheng Y. (2021). Design of polymers for siRNA delivery: Recent progress and challenges. VIEW.

[18] Gavrilov K., Saltzman W.M. (2012). Therapeutic siRNA: Principles, challenges, and strategies. Yale J. Biol. Med..

[19] Kulkarni J.A., Witzigmann D., Chen S., Cullis P.R., van der Meel R. (2019). Lipid Nanoparticle Technology for Clinical Translation of siRNA Therapeutics. Acc. Chem. Res..

[20] Springer A.D., Dowdy S.F. (2018). GalNAc-siRNA Conjugates: Leading the Way for Delivery of RNAi Therapeutics. Nucleic Acid Ther..

[21] Levanova A., Poranen M.M. (2018). RNA Interference as a Prospective Tool for the Control of Human Viral Infections. Front. Microbiol..

[22] Drew L. (2019). Why rare genetic diseases are a logical focus for RNA therapies. Nature.

[23] Mahima K., Rddhima R., Shobhit M. (2020). Exploring Promises of siRNA in Cancer Therapeutics. Curr Cancer Ther Rev..

[24] Uludağ H., Parent K., Aliabadi H.M., Haddadi A. (2020). Prospects for RNAi Therapy of COVID-19. Front. Bioeng. Biotechnol..

[25] Sajid M.I., Moazzam M., Cho Y., Kato S., Xu A., Way J.J., Lohan S., Tiwari R.K. (2021). siRNA Therapeutics for the Therapy of COVID-19 and Other Coronaviruses. Mol. Pharm..

[26] Fitzgerald K., White S., Borodovsky A., Bettencourt B.R., Strahs A., Clausen V., Wijngaard P., Horton J.D., Taubel J., Brooks A. (2017). A Highly Durable RNAi Therapeutic Inhibitor of PCSK9. N. Engl. J. Med..

[27] Kim Y.-K. (2020). RNA Therapy: Current Status and Future Potential. Chonnam. Med. J..

[28] Jeong J.H., Mok H., Oh Y.-K., Park T.G. (2009). siRNA Conjugate Delivery Systems. Bioconjug. Chem..

[29] Kaiser P.K., Symons R.C.A., Shah S.M., Quinlan E.J., Tabandeh H., Do D.V., Reisen G., Lockridge J.A., Short B., Guerciolini R. (2010). RNAi-based treatment for neovascular age-related macular degeneration by Sirna-027. Am. J. Ophthalmol..

[30] Chakraborty C., Sharma A.R., Sharma G., Doss C.G.P., Lee S.-S. (2017). Therapeutic miRNA and siRNA: Moving from Bench to Clinic as Next Generation Medicine. Mol. Ther. Nucleic Acids.

[31] Adams D., Gonzalez-Duarte A., O’Riordan W.D., Yang C.-C., Ueda M., Kristen A.V., Tournev I., Schmidt H.H., Coelho T., Berk J.L. (2018). Patisiran, an RNAi Therapeutic, for Hereditary Transthyretin Amyloidosis. N. Engl. J. Med..

[32] Hoy S.M. (2018). Patisiran: First Global Approval. Drugs.

[33] Anderson K.E. (2019). Acute hepatic porphyrias: Current diagnosis & management. Mol. Genet. Metab..

[34] Scott L.J. (2020). Givosiran: First Approval. Drugs.

[35] Agarwal S., Simon A.R., Goel V., Habtemariam B.A., Clausen V.A., Kim J.B., Robbie G.J. (2020). Pharmacokinetics and Pharmacodynamics of the Small Interfering Ribonucleic Acid, Givosiran, in Patients With Acute Hepatic Porphyria. Clin. Pharmacol. Ther..

[36] Wang B. (2021). Novel treatment options for acute hepatic porphyrias. Curr. Opin. Gastroenterol..

[37] Garrelfs S.F., Frishberg Y., Hulton S.A., Koren M.J., O’Riordan W.D., Cochat P., Deschênes G., Shasha-Lavsky H., Saland J.M., van’t Hoff W.G. (2021). Lumasiran, an RNAi Therapeutic for Primary Hyperoxaluria Type 1. N. Engl. J. Med..

[38] Scott L.J., Keam S.J. (2021). Lumasiran: First Approval. Drugs.

[39] Lamb Y.N. (2021). Inclisiran: First Approval. Drugs.

[40] German C., Shapiro M. (2019). Small Interfering RNA Therapeutic Inclisiran: A New Approach to Targeting PCSK9. BioDrugs.

[41] Raal F.J., Kallend D., Ray K.K., Turner T., Koenig W., Wright R.S., Wijngaard P.L., Curcio D., Jaros M.J., Leiter L.A. (2020). Inclisiran for the Treatment of Heterozygous Familial Hypercholesterolemia. N. Engl. J. Med..

[42] Leqvio (inclisiran) for the Treatment of Hypercholaesterolemia. https://www.clinicaltrialsarena.com/projects/leqvio-inclisiran-hypercholesterolaemia.

[43] Elbashir S.M., Harborth J., Lendeckel W., Yalcin A., Weber K., Tuschl T. (2001). Duplexes of 21-nucleotide RNAs mediate RNA interference in cultured mammalian cells. Nature.

[44] Caplen N.J., Parrish S., Imani F., Fire A., Morgan R.A. (2001). Specific inhibition of gene expression by small double-stranded RNAs in invertebrate and vertebrate systems. Proc. Natl. Acad. Sci. USA.

[45] Tabernero J., Shapiro G.I., LoRusso P.M., Cervantes A., Schwartz G.K., Weiss G.J., Paz-Ares L., Cho D.C., Infante J.R., Alsina M. (2013). First-in-humans trial of an RNA interference therapeutic targeting VEGF and KSP in cancer patients with liver involvement. Cancer Discov..

[46] Schwarz D.S., Ding H., Kennington L., Moore J.T., Schelter J., Burchard J., Linsley P.S., Aronin N., Xu Z., Zamore P.D. (2006). Designing siRNA That Distinguish between Genes That Differ by a Single Nucleotide. PLoS Genet..

[47] Pei Y., Tuschl T. (2006). On the art of identifying effective and specific siRNAs. Nat. Methods.

[48] Reynolds A., Leake D., Boese Q., Scaringe S., Marshall W.S., Khvorova A. (2004). Rational siRNA design for RNA interference. Nat. Biotechnol..

[49] Fakhr E., Zare F., Teimoori-Toolabi L. (2016). Precise and efficient siRNA design: A key point in competent gene silencing. Cancer Gene Ther..

[50] Tilli T.M., da Silva Castro C., Tuszynski J.A., Carels N. (2016). A strategy to identify housekeeping genes suitable for analysis in breast cancer diseases. BMC Genomics.

[51] Chou Y.-C., Lai M.M., Wu Y.-C., Hsu N.-C., Jeng K.-S., Su W.-C. (2015). Variations in genome-wide RNAi screens: Lessons from influenza research. J. Clin. Bioinform..

[52] Scarborough R.J., Gatignol A. (2017). RNA Interference Therapies for an HIV-1 Functional Cure. Viruses.

[53] Sundara Rajan S., Ludwig K.R., Hall K.L., Jones T.L., Caplen N.J. (2020). Cancer biology functional genomics: From small RNAs to big dreams. Mol. Carcinog..

[54] Davis M.E., Zuckerman J.E., Choi C.H.J., Seligson D., Tolcher A., Alabi C.A., Yen Y., Heidel J.D., Ribas A. (2010). Evidence of RNAi in humans from systemically administered siRNA via targeted nanoparticles. Nature.

[55] Grzelinski M., Urban-Klein B., Martens T., Lamszus K., Bakowsky U., Höbel S., Czubayko F., Aigner A. (2006). RNA Interference-Mediated Gene Silencing of Pleiotrophin Through Polyethylenimine-Complexed Small Interfering RNAs In Vivo Exerts Antitumoral Effects in Glioblastoma Xenografts. Hum. Gene Ther..

[56] Chow M.Y.T., Qiu Y., Lam J.K.W. (2020). Inhaled RNA Therapy: From Promise to Reality. Trends Pharmacol Sci..

[57] Li S.-D., Huang L. (2009). Nanoparticles evading the reticuloendothelial system: Role of the supported bilayer. Biochim. Biophys. Acta BBA Biomembr..

[58] Mo R., Gu Z. (2016). Tumor microenvironment and intracellular signal-activated nanomaterials for anticancer drug delivery. Mater. Today.

[59] Meng Z., Lu M. (2017). RNA Interference-Induced Innate Immunity, Off-Target Effect, or Immune Adjuvant?. Front. Immunol..

[60] Marques J.T., Williams B.R.G. (2005). Activation of the mammalian immune system by siRNAs. Nat. Biotechnol..

[61] Juliano R.L., Ming X., Nakagawa O. (2012). Cellular Uptake and Intracellular Trafficking of Antisense and siRNA Oligonucleotides. Bioconjug. Chem..

[62] Yu J.-Y., DeRuiter S.L., Turner D.L. (2002). RNA interference by expression of short-interfering RNAs and hairpin RNAs in mammalian cells. Proc. Natl. Acad. Sci. USA.

[63] Pusch O., Boden D., Silbermann R., Lee F., Tucker L., Ramratnam B. (2003). Nucleotide sequence homology requirements of HIV-1-specific short hairpin RNA. Nucleic Acids Res..

[64] Mansoori B., Mohammadi A., Shir Jang S., Baradaran B. (2016). Mechanisms of immune system activation in mammalians by small interfering RNA (siRNA). Artif. Cells Nanomed. Biotechnol..

[65] Robbins M., Judge A., MacLachlan I. (2009). siRNA and Innate Immunity. Oligonucleotides.

[66] Freeley M., Long A. (2013). Advances in siRNA delivery to T-cells: Potential clinical applications for inflammatory disease, cancer and infection. Biochem. J..

[67] Mantei A., Rutz S., Janke M., Kirchhoff D., Jung U., Patzel V., Vogel U., Rudel T., Andreou I., Weber M. (2008). siRNA stabilization prolongs gene knockdown in primary T lymphocytes. Eur. J. Immunol..

[68] Simon B., Harrer D.C., Schuler-Thurnefr B., Schaft N., Schuler G., Dörrie J., Uslu U. (2018). The siRNA-mediated downregulation of PD-1 alone or simultaneously with CTLA-4 shows enhanced in vitro CAR-T-cell functionality for further clinical development towards the potential use in immunotherapy of melanoma. Exp. Dermatol..

[69] Whitehead K.A., Langer R., Anderson D.G. (2009). Knocking down barriers: Advances in siRNA delivery. Nat. Rev. Drug Discov..

[70] Alexis F., Pridgen E., Molnar L.K., Farokhzad O.C. (2008). Factors Affecting the Clearance and Biodistribution of Polymeric Nanoparticles. Mol. Pharm..

[71] Ilinskaya A.N., Dobrovolskaia M.A. (2016). Understanding the immunogenicity and antigenicity of nanomaterials: Past, present and future. Toxicol. Appl. Pharmacol..

[72] Elnemr A., Ohta T., Yachie A., Fushida S., Ninomiya I., Nishimura G.I., Yamamoto M., Ohkuma S., Miwa K. (2000). N-ethylmaleimide-enhanced phosphatidylserine externalization of human pancreatic cancer cells and immediate phosphatidylserine-mediated phagocytosis by macrophages. Int. J. Oncol..

[73] Kaul G., Amiji M. (2002). Long-Circulating Poly(Ethylene Glycol)-Modified Gelatin Nanoparticles for Intracellular Delivery. Pharm. Res..

[74] Bhattacharya S. (2021). Fabrication of poly(sarcosine), poly (ethylene glycol), and poly (lactic-co-glycolic acid) polymeric nanoparticles for cancer drug delivery. J. Drug Deliv. Sci. Technol..

[75] Hui Y., Yi X., Wibowo D., Yang G., Middelberg A.P.J., Gao H., Zhao C.X. (2020). Nanoparticle elasticity regulates phagocytosis and cancer cell uptake. Sci. Adv..

